# ﻿*Androctonus tihamicus* sp. nov. from the Mecca Province, Saudi Arabia (Scorpiones, Buthidae)

**DOI:** 10.3897/zookeys.1152.101100

**Published:** 2023-03-02

**Authors:** Abdulaziz R. Alqahtani, Ersen Aydın Yağmur, Ahmed Badry

**Affiliations:** 1 Department of Biology, College of Science, University of Bisha, P.O. Box 551, Bisha 61922, Saudi Arabia University of Bisha Bisha Saudi Arabia; 2 Alaşehir Vocational School, Manisa Celal Bayar University, Manisa, Turkey Manisa Celal Bayar University Manisa Turkiye; 3 Department of Zoology, Faculty of Science, Al Azhar University, Nasr City, P.O. Box: 11751, Cairo, Egypt Al Azhar University Cairo Egypt

**Keywords:** Molecular phylogeny, mtDNA, new species, scorpion, Tihamah Plain

## Abstract

We describe and illustrate a new scorpion species, *Androctonus tihamicus* **sp. nov.**, from the Mecca Province of southwestern Saudi Arabia. The new species is compared to the genus *Androctonus* Ehrenberg, 1828, which is distributed throughout the Middle East, and especially to *A. australis* (Linnaeus, 1758). We provide the molecular phylogeny for this species.

## ﻿Introduction

*Androctonus* Ehrenberg, 1828 is one of the largest and most widely distributed genera of the family Buthidae (order Scorpiones) in North Africa, the Middle East, and western Asia (Fet and Lowe 2000). The genus has 36 valid species (Rein 2023), of which most species are known to deliver severe stings. *Androctonus* is also very taxonomically confused due to great intraspecific morphological and molecular variation (Ben Ali et al. 2000; Lourenço 2005; Lourenço and Qi 2006, 2007; Lourenço 2008; Othmen et al. 2009; Alqahtani et al. 2022a, b). As already shown by Hendrixson (2006) and Alqahtani et al. (2019), several *Androctonus* species are widely distributed in Saudi Arabia, where they inhabit diverse habitats. Alqahtani et al. (2022c) found intraspecific variation among populations of *A. crassicauda* (Olivier, 1807) from different regions of Saudi Arabia and suggested the existence of cryptic taxa. Also, Hendrixson (2006) noted light-coloured *A. crassicauda*, and Alqahtani et al. (2019) questioned the existence of *A. australis* (Linnaeus, 1758) in Saudi Arabia.

In this study, we examined a *Androctonus* population from Mecca Province, which had been previously reported as *A. crassicauda* (Vachon 1979; Hendrixson 2006). We describe *A. tihamicus* sp. nov. and compare this new species with specimens of *A. australis* from Egypt (Sinai) and other *Androctonus* species from the Middle East. Additionally, we provide the first molecular phylogeny, supplemented by morphological comparisons, of the genus *Androctonus* in Saudi Arabia.

## ﻿Materials and methods

We collected 21 specimens of *A. tihamicus* sp. nov. at night using ultraviolet light in Mecca Province between 1 September 2018 and 29 January 2022. The specimens were preserved in 96% alcohol. Photographs were taken as described by Yağmur (2021). The trichobothrial nomenclature follows Vachon (1974), and the morphological nomenclature follows Francke (1977), Stahnke (1971), and Hjelle (1990). The male holotype and a female paratype of *A. tihamicus* sp. nov. are deposited at Alaşehir Zoological Museum, Manisa Celal Bayar University, Alaşehir, Manisa, Turkey (**AZMM**) and at the Al-Azhar University Zoological Collection (**AUZC**), Nasr City, Cairo, Egypt.

### ﻿Molecular analysis

The whole genomic DNA was isolated from four freshly preserved scorpion specimens using Qiagen DNA extraction kits following the manufacturer’s instructions. The amplified 16S rRNA gene products were checked and purified (Qiagen) according to the manufacturer’s instructions. A fragment of the 16S rRNA gene was amplified via a standard polymerase chain reaction (PCR) using the invertebrate universal primers, as determined and sequenced on an ABI 3500 automated sequencer (Applied Biosystems Inc., USA) and following Gantenbein et al. (1999). The chromatograms and sequences were examined and edited using BioEdit v. 7.2.5 (Hall 1999). Additional sequence data were retrieved from GenBank for *A. amoreuxi* (Audouin, 1825), *A. australis*, and *A. crassicauda* from Egypt, Iran, Turkey, and Saudi Arabia. A sequence of *Scorpio palmatus* Linnaeus, 1758 (AY156570.1) was downloaded as the outgroup. Sequence data were edited using Mega 6 (Tamura et al. 2013) and aligned using the default settings of ClustalW. Nucleotide composition was calculated from the ingroup sequences only. The genetic distances (*p*-distances) were calculated for the entire data set using Mega 6 (Tamura et al. 2013). Phylogenetic analyses of the 16S data set (*n* = 35) were performed as proposed by Alqahtani and Badry (2020). Maximum-parsimony and neighbor-joining analyses were conducted with Paup v. 4 (Swofford 2003) combined with heuristic clustering based on TBR (tree bisection and reconnection) branch swapping. A character was considered missing when a gap was present in an alignment. In addition to 1000 bootstrapping replicates, random additions of taxa were used to assess the degree of confidence within the nodes (Felsenstein 2002). The best-fit nucleotide evolution models were preferred using Paup v. 4 (Swofford 2003) and MrModeltest v. 2.3 (Nylander 2004) based on the Akaike Information Criterion (Akaike 1973). To infer the geographic structure, MrBayes v. 3.1.2 (Ronquist et al. 2012) was used to implement Bayesian inference. The analyses were executed for a million generations, and output parameters were plotted with Tracer v. 1.7 (Rambaut et al. 2018).

## ﻿Systematics

### ﻿Family Buthidae C.L. Koch, 1837

**Genus *Androctonus* Ehrenberg, 1828**

#### ﻿ Androctonus tihamicus sp. nov.

Taxon classificationAnimaliaScorpionesButhidae

800E6DE4-A6BC-5C6C-8AB7-989F52189F01

https://zoobank.org/1E854848-8838-4EB8-807A-611AFD4A8E39

Figs 1, 2, 3, 4, 5, 6, 7, 8, 9, 10, 11, 12, 13, 14, 15; Table 1

Buthus australis citrina (incorrect spelling)–Gough and Hirst 1927: 4.
Androctonus crassicauda –Vachon 1979: 31–34, figs 1, 2, 4.
Androctonus australis –Levy and Amitai 1980: 36, 40; Alqahtani et al. 2019: 21: fig. 2a ; Al-Asmari et al. 2013: fig. 7.
Androctonus crassicauda –Hendrixson 2006: 38–43, figs 1, 2, pl. 1.
Androctonus amoreuxi –Al-Asmari et al. 2013: table 1.

##### Type materials.

***Holotype*** ♂: **Saudi Arabia**, Mecca Province, Al-Gunfuda, 1.xi.2018, 19.166389°N, 41.099806°E, 10 m a.s.l., Alqahtani A.R. leg. (AZMM/Sco-2018:01). ***Paratypes*** (10 ♀, 10 ♂): **Saudi Arabia**, Mecca Province, Al-Gunfuda, 19.1674°N, 41.0999°E, 8 m a.s.l., 1.xi.2018, 1♀, Alqahtani A.R. leg. (AZMM/Sco-2018:02). Mecca, Province, Al Baydayn, 19.1836°N, 41.2334°E 45 m a.s.l., xi.2018, 2♀, 1♂, Alqahtani A.R. leg. (AUZC/Sco-2018:3-5). Mecca Province, Keyad, xi.2018, 18°42'00.1"N 41°24'00.4"E, 40 m a.s.l., 1♀, 1♂, Alqahtani A.R. & Badry A. leg. (AUZC/Sco-2018:6-7). Mecca Province, Al-Gunfuda, 19.1674°N, 41.0999°E, 8 m a.s.l., 4.I.2022, 6♀, 8♂, Alqahtani A.R. & Badry A. leg. (AUZC/Sco-2022:8-21).

**Table 1.** Comparative measurements of types of *Androctonus tihamicus* sp. nov. Abbreviations: length (L), width (W; for carapace, it corresponds to posterior width), depth (D).

Dimensions (mm)		*A. tihamicus* sp. nov.	*A. tihamicus* sp. nov.
		♂, holotype	♀, paratype
Carapace	L / W	8.79 / 8.91	10.36 / 10.38
Mesosoma	L	17.05	21.49
Tergite VII	L / W	5.36 / 9.68	5.97 / 11.13
Metasoma + telson	L	45.28	52.66
Segment I	L / W / D	5.68 / 6.21 / 5.29	6.08 / 6.10 / 5.73
Segment II	L / W / D	7.14 / 6.68 / 6.23	7.54 / 6.69 / 6.14
Segment III	L / W / D	7.25 / 7.08 / 6.59	8.46 / 7.25 / 6.76
Segment IV	L / W / D	9.34 / 7.38 / 6.61	10.02 / 7.28 / 6.74
Segment V	L / W / D	9.23 / 6.65 / 4.93	10.75 / 6.92 / 5.46
Telson	L / W / D	7.14 / 3.50 / 2.56	9.79 / 4.16 / 3.13
Pedipalp	L	31.34	34.31
Femur	L / W	7.49 / 2.61	7.96 / 2.84
Patella	L / W	8.89 / 3.66	9.58 / 4.09
Chela	L	14.96	17.39
Manus	L / W / D	5.1/ 4.82 / 4.87	5.88 / 4.36 / 5.26
Movable finger	L	9.60	11.50
Fixed finger	L	7.71	9.40
**Total**	**L**	**71.82**	**84.51**

**Figure 1.**
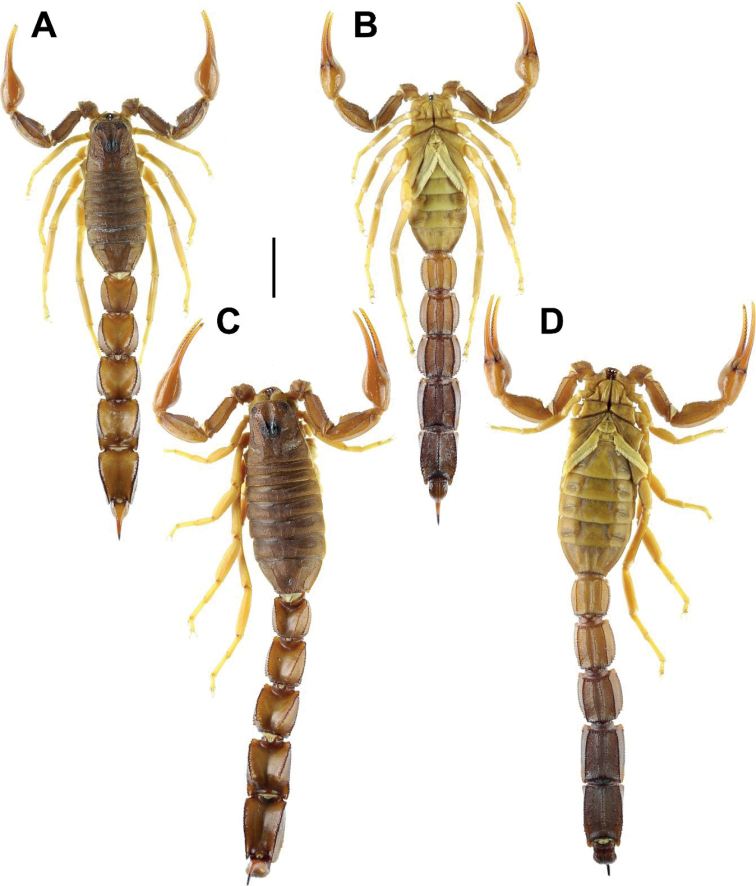
Habitus of *Androctonus tihamicus* sp. nov., male holotype and female paratype **A** male in dorsal view **B** male in ventral view **C** female in dorsal view **D** female in ventral view. Scale bar: 10 mm.

##### Comparative material.

*Androctonus australis*, **Egypt**, Bir El-Abd, north of Sinai Peninsula, 31.0142°N, 32.7486°E, 1♀, 1♂ (AZMM/Sco-2003:03-04).

**Figure 2.**
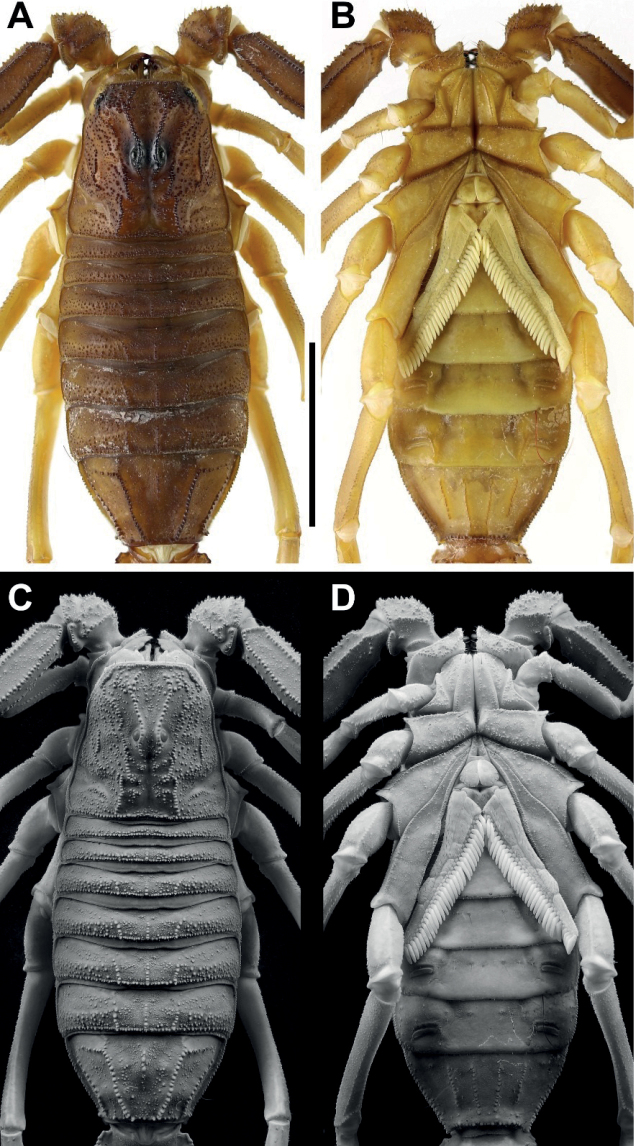
*Androctonus tihamicus* sp. nov., male holotype **A, C** carapace and mesosoma **B, D** sternopectinal area and ventral of mesosoma (**A, B** under white light; **C, D** under UV light). Scale bar: 10 mm.

##### Etymology.

The new species is named after the Tihamah Plain, the coastal plain along the Red Sea.

**Figure 3.**
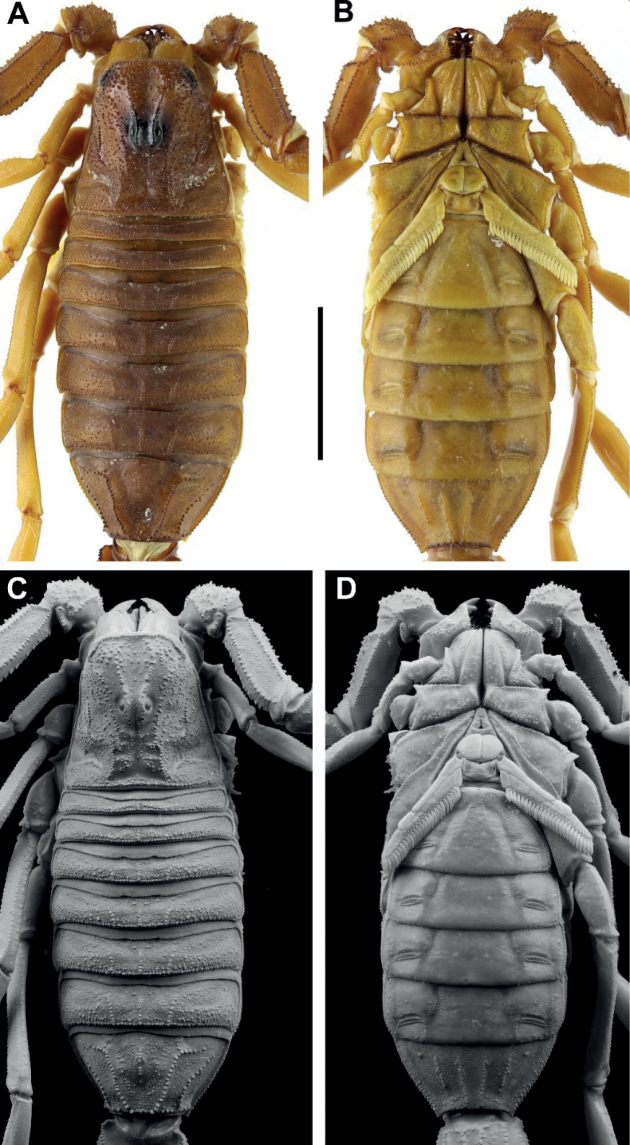
*Androctonus tihamicus* sp. nov., female paratype **A, C** carapace and mesosoma **B, D** sternopectinal area and ventral of mesosoma (**A, B** under white light; **C, D** under UV light). Scale bar: 10 mm.

##### Diagnosis.

Medium-sized scorpion with average length 76.15 mm in females and 77.06 mm in males. General color light brown to reddish brown; chela reddish yellow. Legs completely yellow, without any spots in both males and females. Fixed and movable fingers with 13–15 (mostly 14) and 13–15 (mostly 14) principal rows of denticles, respectively. Carapace coarsely granulose; granules at anterior corners larger. Posterior median and central median carinae coarsely granulose and strong. Ventrolateral carinae of metasomal segment V moderately developed, with granules gradually and slightly increasing in size posteriorly. Dorsolateral carinae of segments III–IV strong, with large, serrate, gradually increasing in size granules posteriorly and two large granules posteriorly. Dorsolateral carinae of metasomal segment V with rounded, distinct, large granules anteriorly, and without granules posteriorly. Pectines with 31–33 teeth in males and 23–31 in females.

**Figure 4.**
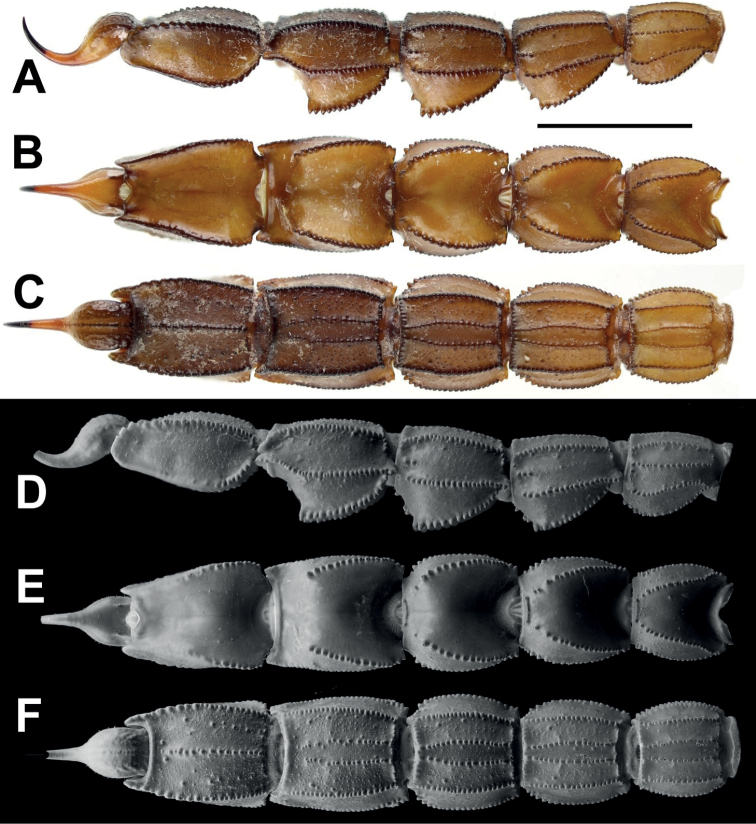
*Androctonus tihamicus* sp. nov., metasoma and telson of male holotype **A, D** lateral view **B, E** dorsal view **C, F** ventral view (**A–C** under white light; **D–F** under UV light). Scale bar: 10 mm.

##### Description based on holotype.

***Coloration***: general color light brown to reddish brown. Prosoma: carapace reddish brown; carinae and surrounds of eyes marked by black pigmentation. Mesosoma: reddish brown, slightly lighter than carapace. Metasoma: segments I–V light brown, ventral surfaces of reddish brown; carinae marked with brown or black pigmentation; vesicle reddish brown anteriorly, light brown posteriorly; aculeus reddish at base and blackish at extremity. Venter yellowish to reddish yellow; pectines pale yellow. Chelicerae yellowish, without variegated spots in male and with diffused variegated spots in females; fingers yellowish, with dark teeth. Pedipalps: femur and patella brownish yellow, with dark reddish brown carinae; chela reddish brown, fingers reddish brown but dark yellow posteriorly, denticles black. Legs uniformly dark yellow, without spots.

**Figure 5.**
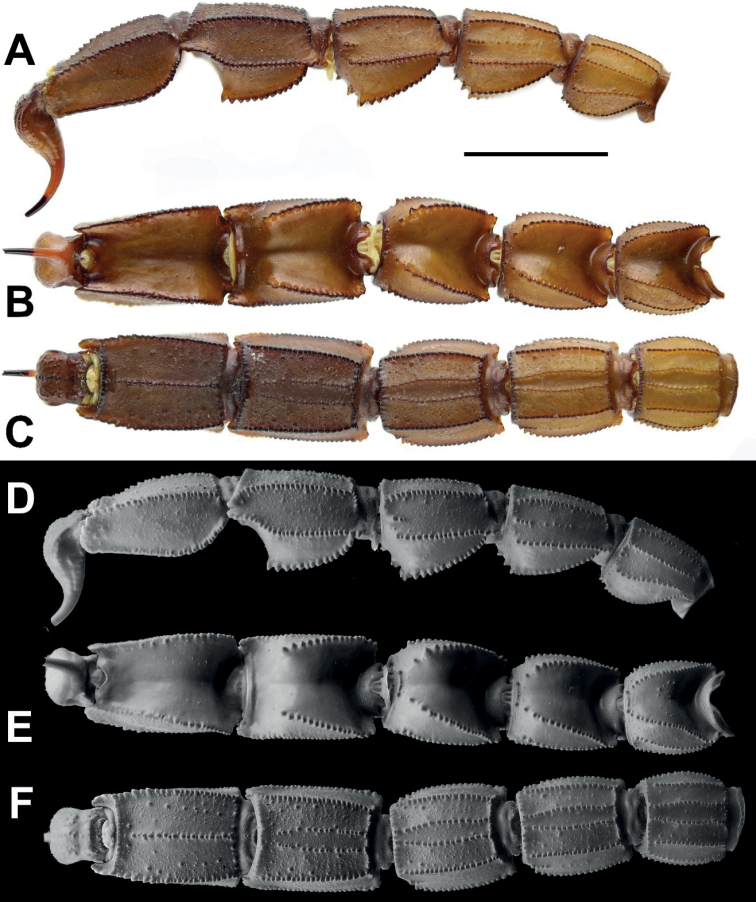
*Androctonus tihamicus* sp. nov., metasoma and telson of female paratype **A, D** lateral view **B, E** dorsal view **C, F** ventral view (**A–C** under white light; **D–F** under UV light). Scale bar: 10 mm.

***Prosoma***: carapace trapezoidal, wider than long; all carinae strong and coarsely granular. Larger, anterior and posterior median and central median carinae coarsely granulose; strong intergranular area with medium-sized and large granules, anteriorly with very large granules; anterior margin nearly straight, with some stout macrosetae; all furrows moderate to weak; median ocular tubercle slightly anterior to center of carapace; eyes separated by two ocular diameters; five pairs of lateral eyes, first three pairs of moderate size and aligned, last two pairs vestigial; sternum triangular, narrow, slightly longer than wide. Cheliceral dentition typical for genus, as defined by Vachon (1963); surface smooth, with coarse granules arranged in longitudinal ridges.

**Figure 6.**
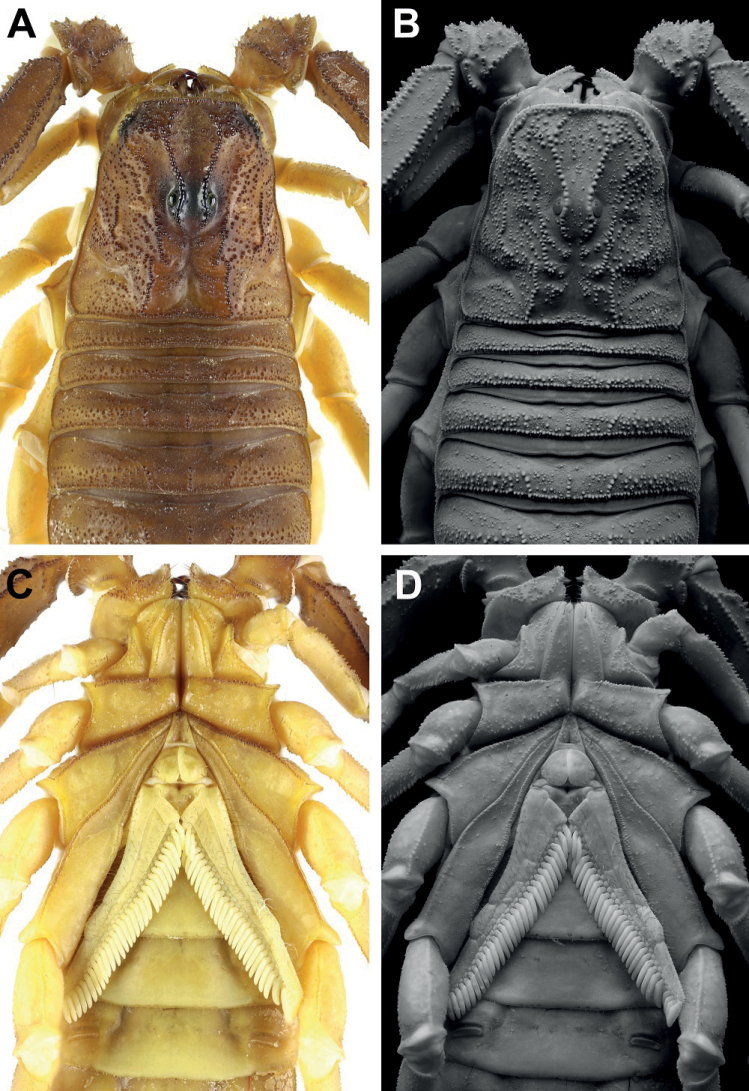
*Androctonus tihamicus* sp. nov., male holotype **A, B** carapace **C, D** sternopectinal area (**A, C** under white light; **B, D** under UV light).

***Mesosoma***: tergites densely granular; pretergites finely granular, posttergites coarsely granular; posterior margins with a row of distinct strong granules; I–VI with three moderate to strong, granulose carinae (median and submedians), projecting beyond posterior margin. Tergite VII pentacarinate, with scattered fine granules (median, submedians, and laterals). Venter: sternum standard for the genus: type 1, triangular; genital operculum divided longitudinally, forming two semi-oval plates; pectines long, reaching leg IV coxa/trochanter joint, narrow, densely setose; tooth count 31/33; basal plate heavily sclerotized and wider than long, with anterior margin with strong, median indentation and posterior margin widely convex. Sternites sparsely setose, without granules, smooth with very elongated spiracles and slit-like without granulation; sternites III–VI carinate, with two vestigial furrows; sternite VI with fine, very scattered granules; sternite III without carinae; sternite VII with two pairs of strong granular carinae.

**Figure 7.**
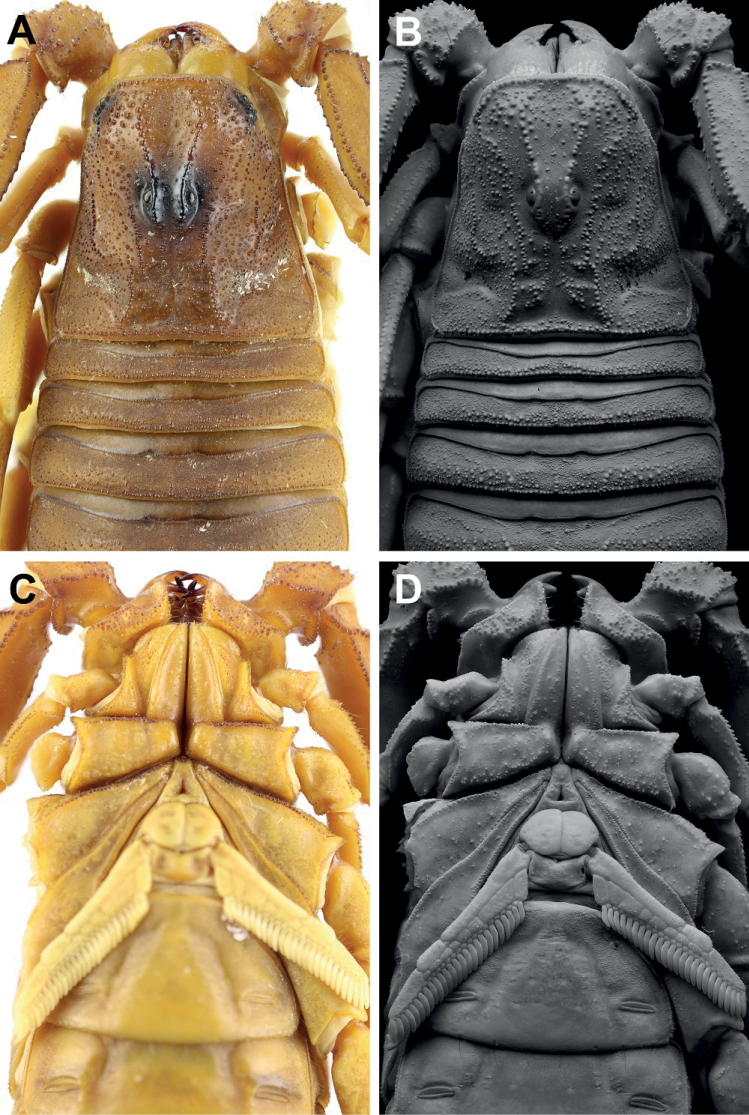
*Androctonus tihamicus* sp. nov., female paratype **A, B** carapace **C, D** sternopectinal area (**A, C** under white light; **B, D** under UV light).

***Metasoma***: very sparsely setose, with all segments robust. All segments longer than wide; segments I–III very slightly longer than wide; wider than deep; inter-carinal tegument of dorsal surface without granulation and smooth, lateral surface slightly roughened, with scattered fine and moderately sparse granules, ventral surface rough with moderately dense fine granules and scattered large granules on segments I–V; dorsal furrow moderately deep and wide on all segments; segment I with 10 carinae, lateral infra-median carinae complete and moderate, segment II with 10 carinae, lateral infra-median incomplete, present on posterior quarter, strongly granular, with three granules; segment III with 10 carinae, lateral infra-median incomplete with two granules; segment IV with eight, and segment V with five carinae. Dorsolateral carinae of segments I–IV strong, with serrate granules that gradually increase posteriorly. Granules small on segment I, moderate on segment II, and large on segments III–IV. Segment V with strong, rounded carinae, posteriorly smooth with very rounded, shallow granules anteriorly. Lateral supra-median carinae strong on segment I–IV, with moderate, rounded and crenulate granules on segment I, large, rounded, crenulate granules on II–V, and more swollen and one very large, rounded granule at the posterior end of segment IV. Ventrolateral carinae on segments I–IV strong, with large, rounded granules; strong with gradually and slightly increased granules posteriorly on segment V. Ventral submedian carinae moderate on segments I–V, with moderately rounded granules. Anal arch laterally with three rounded lobes, the inferior lobe twice as large as the other two lobes. Telson slender, elongate, densely setose; vesicle small somewhat globose, tegument glossy and essentially smooth, with only some coarse granules and a coarse but very poorly defined ventro-median carinae; setal pair subaculear; aculeus very long and thick, as long as vesicle, and moderately curved.

**Figure 8.**
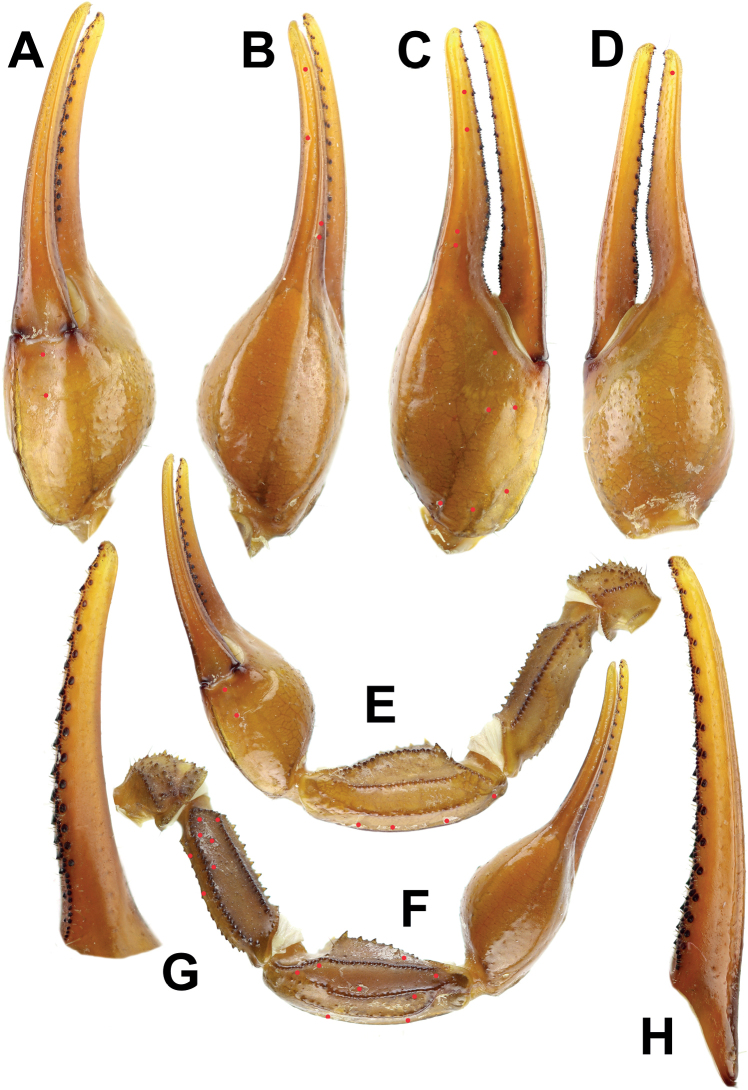
*Androctonus tihamicus* sp. nov., male holotype **A** ventral view of chela **B** dorsal view of chela **C** external view of chela **D** internal view of chela **E** ventral view of pedipalp **F** dorsal view of pedipalp **G** fixed finger dentition **H** movable finger dentition (trichobothrial pattern is indicated by red circles).

***Legs***: long, slender, covered by several macrosetae. Basitarsus of legs I–III bear bristle combs; basitarsus of leg IV without bristle comb. Proventral and retroventral basitarsal (pedal) spurs present but tibial spurs present on legs III and IV. Tarsus of legs I–IV ventrally with spine-like setae arranged in two rows: tarsal spurs basally bifurcate, bearing 1–3 macrosetae (four on legs I–II, eight or nine on leg III–IV). Basitarsus of legs I–III with bristle combs; basitarsus of legs IV without bristle combs.

**Figure 9.**
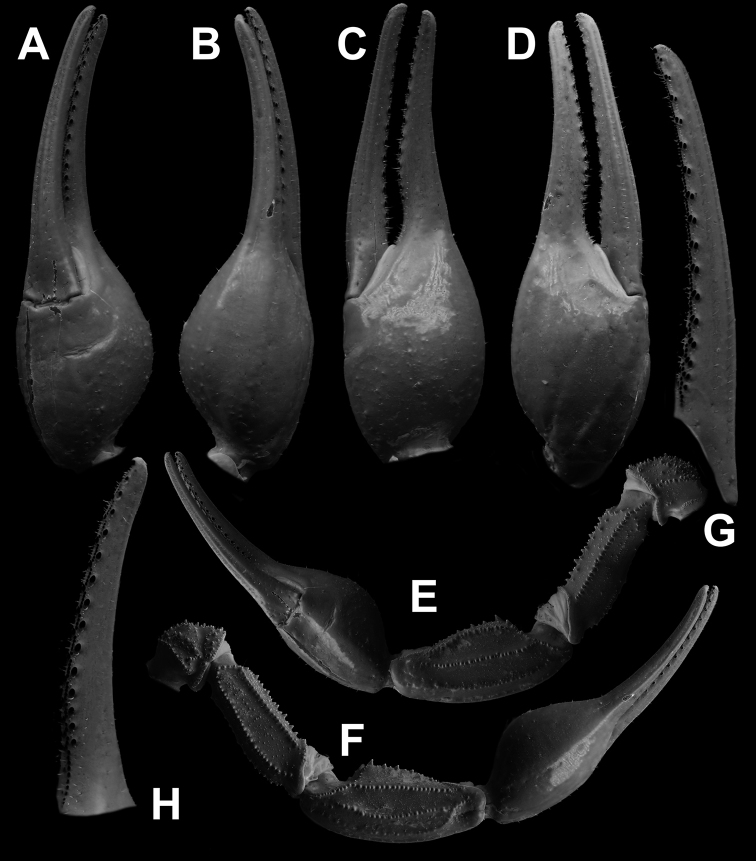
*Androctonus tihamicus* sp. nov., male holotype, under UV light **A** ventral view of chela **B** dorsal view of chela **C** internal view of chela **D** external view of chela **E** ventral view of pedipalp **F** dorsal view of pedipalp **G** fixed finger dentition **H** movable finger dentition.

***Pedipalps***: stocky, moderately long, densely setose. Type A trichobothrial pattern; orthobothriotaxic. Dorsal trichobothria of femur arranged in beta-configuration with d_2_ situated on dorsal surface. Femur pentacarinate, slender, straight; all carinae strong and moderately granulose; intercarinal tegument finely granulose, with irregular, coarse granules dorsally; inner surface with a few coarse granules; dorso-internal carinae with distinct spinoid granules. Patella with seven carinae, moderately slender and straight; surfaces smooth with scattered fine granules; all carinae moderately strong, dorsomedian, dorsoexternal, ventromedian, ventroexternal, ventrointernal carinae granulose, external smooth, dorsointernal with seven spinoid granules; dorsointernal and ventrointernal carinae distally terminate at one spinoid granule. Chela smooth, without carinae, stocky. Manus wider than patella (chela width/patella width = 1.13); internal surface of manus with scattered fine granules; fingers moderately elongate (movable finger length/manus length = 1.76), evenly curved. Movable fingers of pedipalps bear 15 rows of granules and external and internal granules; fixed fingers bear 14 rows of granules, with external and internal accessory granules and three distal granules.

**Figure 10.**
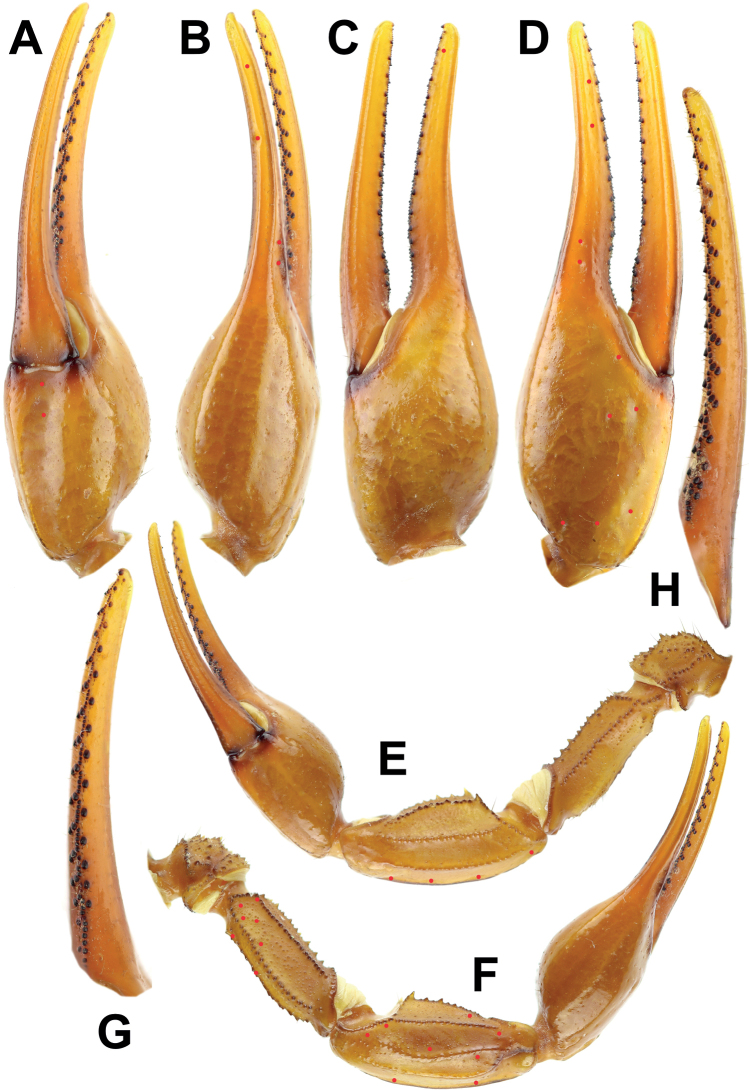
*Androctonus tihamicus* sp. nov, female paratype **A** ventral view of chela **B** dorsal view of chela **C** internal view of chela **D** external view of chela **E** ventral view of pedipalp **F** dorsal view of pedipalp **G** fixed finger dentition **H** movable finger dentition (trichobothrial pattern is indicated by red circles).

**Figure 11.**
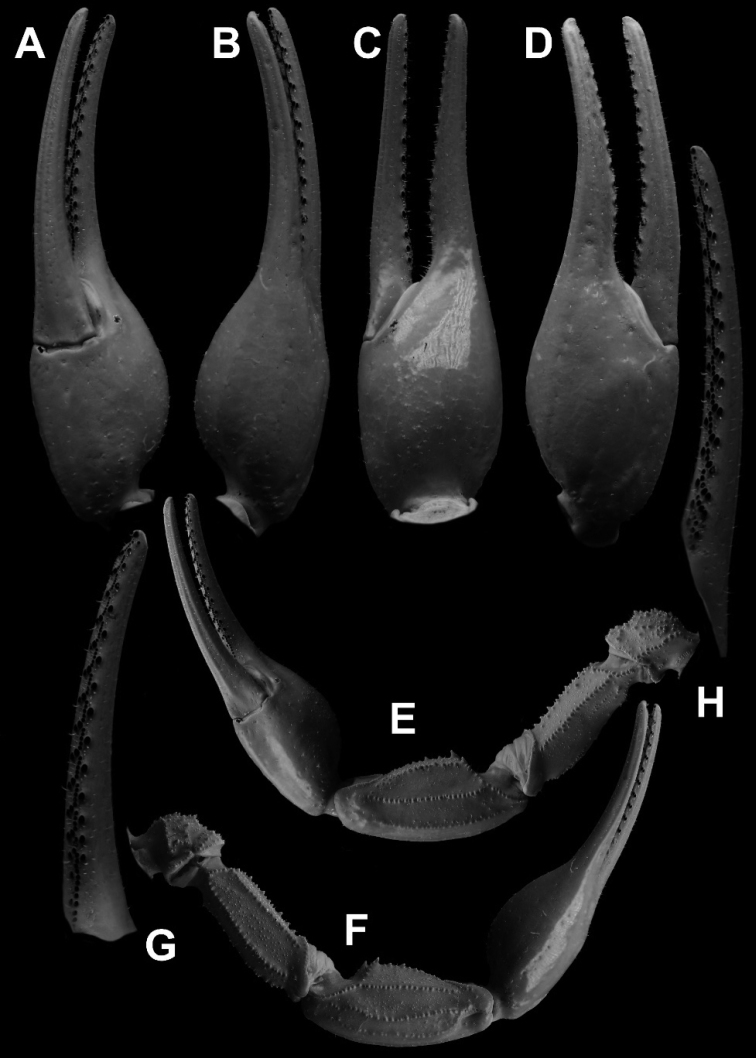
*Androctonus tihamicus* sp. nov., female paratype, under UV light **A** ventral view of chela **B** dorsal view of chela **C** internal view of chela **D** external view of chela **E** ventral view of pedipalp **F** dorsal view of pedipalp **G** fixed finger dentition **H** movable finger dentition.

**Figure 12.**
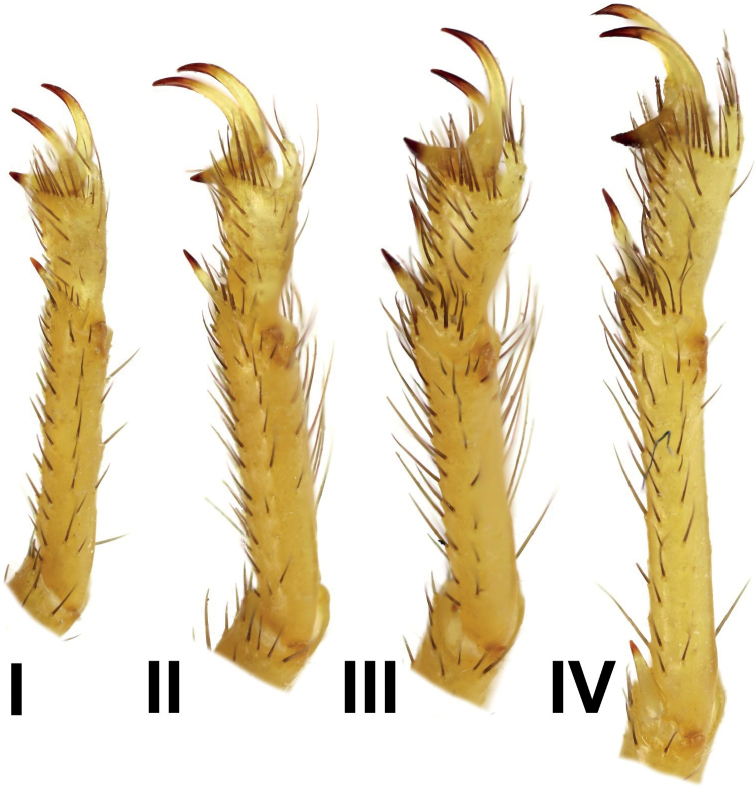
*Androctonus tihamicus* sp. nov., male holotype, right legs I–IV, retrolateral aspect.

##### Comparison.

*Androctonus tihamicus* sp. nov. can be distinguished from other *Androctonus* species in the Middle East by following characters: *A. tihamicus* sp. n. has stocky chela whereas *A. bicolor* Ehrenberg, 1828 has elongate chela. Ventrolateral carinae of 5^th^ segment of *A. tihamicus* sp. nov. lacks large granules and the general color is light to reddish brown, whereas ventrolateral carinae of the 5^th^ segment of *A. turkiyensis* Yağmur, 2021 bears large granules, The general color is brown or black in *A. crassicauda*. The new species has stockier metasoma segments, whereas *A. amoreuxi* (Audouin, 1826) has elongated metasoma segments, and especially the 5^th^ segment, which is remarkably more elongate. The length/width ratios of metasomal segments I–V from the measurements provided by Levy and Amitai (1980) are 0.90-1.12-1.15-1.38-1.71 in males and 0.91-1.12-1.19-1.38-1.71 in females. These ratios in *A. tihamicus* sp. nov. are 0.91-1.06-1.02-1.26-1.38 in males and 0.99-1.12-1.16-1.37-1.55 in females (Table 1). Additionally, *A. amoreuxi* is yellow or olive-yellow. The new species can be distinguished from *A. australis* by the following characteristics: (a) general coloration light to reddish brown with legs uniformly yellow (vs yellow in *A. australis*); (b) pedipalp less robust and movable finger with a small recess and hump (vs more robust and movable finger with a larger recess and hump at base of the fingers in in *A. australis*) (Fig. 14); (c) metasomal segments smaller than *A. australis*; (d) dorsolateral carinae of metasomal segment III has one separate spinoid granule in posterior of segments IV (vs two rounded granules in *A. australis*; Fig. 15, indicated by arrows); (e) ventral carina of 5^th^ metasomal segment without bifurcation (vs bifurcated in *A. australis*); and (f) anal arch with three lateral lobes (vs two lateral lobes in *A. australis*; Fig. 15A, B, E, F).

**Figure 13.**
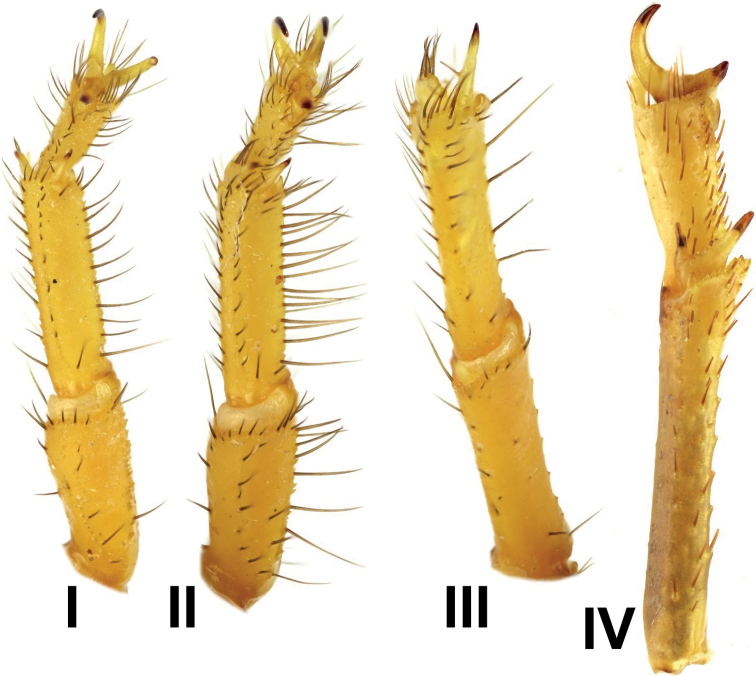
*Androctonus tihamicus* sp. nov., female paratype, right legs I–IV, retrolateral aspect.

**Figure 14.**
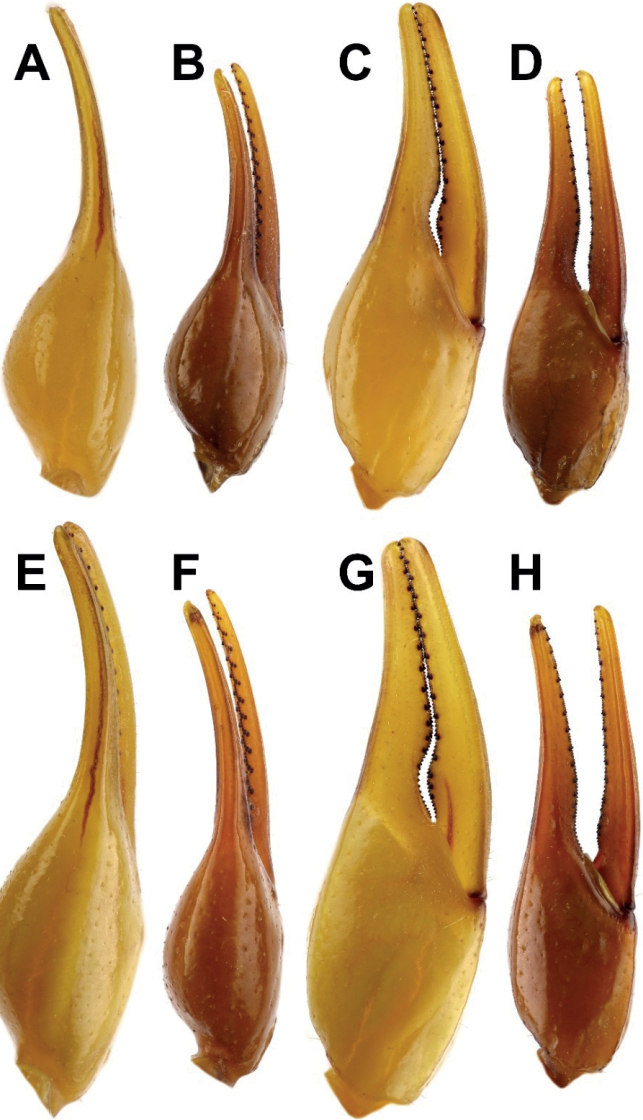
Comparison of chela between male holotype and female paratype of *Androctonus tihamicus* sp. nov. and male and female of *A. australis***A, C, E, G***A. australis***B, D, F, H***A. tihamicus* sp. nov. **A–D** male **E–H** female **A, B, E, F** dorsal **C, D, G, H** external.

**Figure 15.**
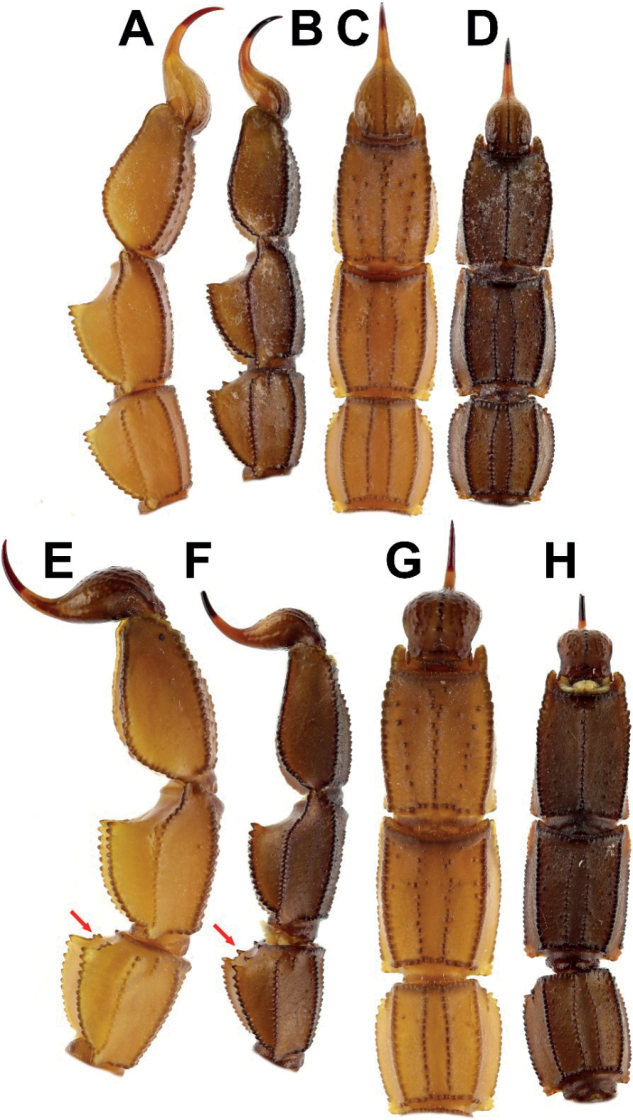
Comparison of metasoma III–V and telson between male holotype and female paratype of *Androctonus tihamicus* sp. nov. and male and female of *A. australis***A, C, E, G***A. australis***B, D, F, H***A. tihamicus* sp. nov. **A–D** male **E–H** female **A, B, E, F** lateral **C, D, G, H** ventral (arrows: see Comparisons in text).

##### Ecology.

*Androctonus tihamicus* sp. nov. is an arenicolous species of scorpion from the Tihamah plain in southwestern Saudi Arabia. The sampling sites are all at low elevations along the coast, where temperatures are high, around 40 °C daily. Southern sites are in the coastal fog desert zone, with high temperatures of 43 °C and a relative humidity of 40–60%.

##### Genetic analysis.

**Figure 16.**
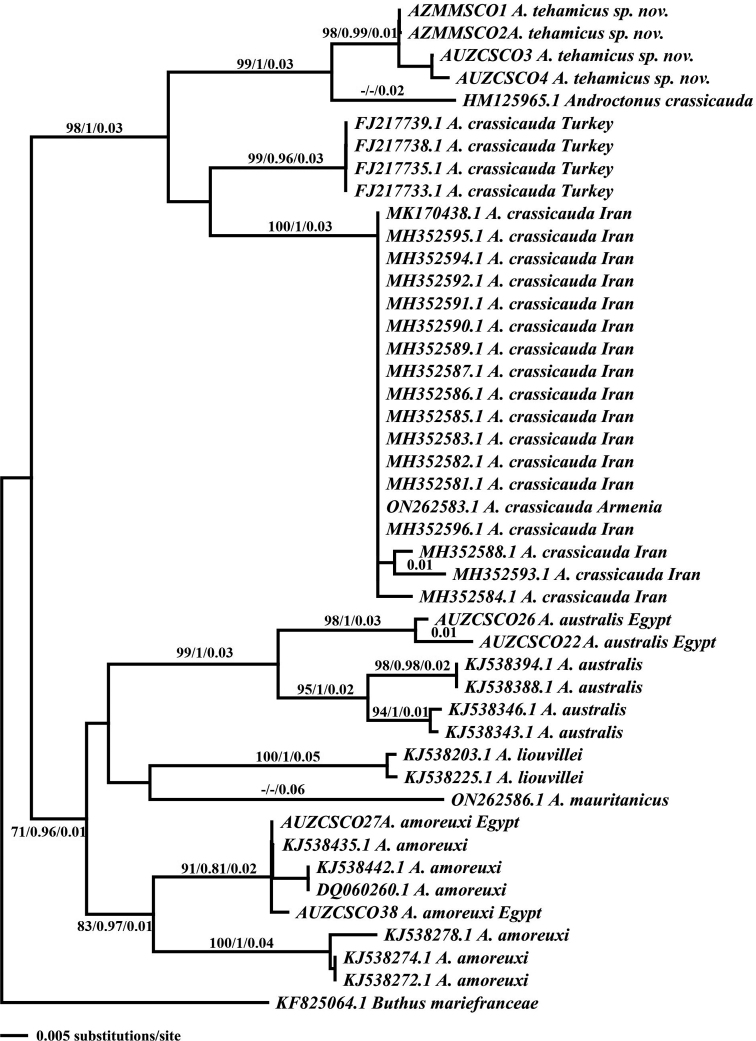
Neighbor-joining (NJ) phylogenetic tree of *Androctonus* species based on 16S rRNA sequences. Numbers above and below branches indicate maximum parsimony bootstrap values/Bayesian posterior probabilities/NJ distance values.

*Androctonus tihamicus* sp. nov. forms a monophyletic clade distinct from *A. crassicauda* (from Saudi Arabia, Turkey, and Iran), and other related *Androctonus* species analyzed (Fig. 16). The new species differs from *A. crassicauda* by a raw genetic distance of 4.0–9.0%, and from *A. amoreuxi* by 13.0%; it differs from *A. australis*, *A. liouvillei* (Pallary, 1924), and *A. mauritanicus* (Pocock, 1902) by a raw genetic distance of 16.0% (Table 2).

**Table 2.** The uncorrected *p*-distance of the sequence divergence of 16S mtDNA sequences between *Androctonus* samples included in this study.

	1	2	3	4	5	6	7	8	9
**1. *A. tihamicus* sp. nov.**		0.01	0.02	0.02	0.02	0.02	0.02	0.02	0.02
**2. *A. crassicauda* Saudi Arabia**	0.04		0.02	0.02	0.02	0.02	0.02	0.02	0.02
**3. *A. crassicauda* Turkey**	0.09	0.09		0.01	0.02	0.02	0.02	0.02	0.02
**4. *A. crassicauda* Iran**	0.09	0.10	0.06		0.02	0.02	0.02	0.02	0.02
**5. *A. amoreuxi***	0.13	0.13	0.13	0.13		0.02	0.02	0.01	0.02
**6. *A. australis***	0.16	0.17	0.15	0.15	0.12		0.02	0.01	0.02
**7. *A. liouvillei***	0.16	0.17	0.13	0.14	0.10	0.12		0.02	0.02
**8. *A. mauritanicus***	0.16	0.17	0.15	0.15	0.12	0.09	0.12		0.02
**9. *Scorpio palmatus* (outgroup)**	0.14	0.15	0.12	0.13	0.12	0.15	0.13	0.14	

## ﻿Discussion

The 16S mitochondrial gene has been successfully used by several authors to delimit cryptic species of *Euscorpius* Thorell, 1876 and *Centruroides* Marx, 1890 (Gantenbein et al. 2001, 2002; Fet et al. 2003, 2016; Ponce-Saavedra et al. 2009; Quijano-Ravell and Ponce-Saavedra 2016). It was for this reason, that we conducted a molecular phylogenetic analysis using the mRNA 16S mitochondrial gene. Our results show a genetic divergence between *A. tihamicus* sp. nov. and populations of *A. crassicauda* from Saudi Arabia, Turkey, and Iran (*p*-distance = 0.04–0.09), and the new species was found to differ from *A. amoreuxi*, *A. australis*, *A. liouvillei* and *A. mauritanicus* by a raw genetic distance of 0.13–0.16 (Table 2). The phylogenetic trees obtained by different models were topologically consistent (Fig. 16). The phylogenetic tree shows that *A. tihamicus* sp. nov. appears most closely related to the *A. crassicauda* sequence from Hail, Saudi Arabia (GeneBank HM125965.1; Desouky and Alshammari 2010). Gough and Hirst (1927) reported *Buthus australis citrina* from Saudi Arabia, an incorrect spelling of the A. (Prionurus) citrinus Ehrenberg, 1828. Its type locality is in Sudan (Dunqulah, Nubia). This taxon was synonymized by Di Caporiacco (1932) with *A. amoreuxi* and the species does not occur in Saudi Arabia. Hendrixson (2006) reported *A. crassicauda* as the only species of *Androctonus* in Saudi Arabia.

Although Levy and Amitai (1980) reported *A. australis* from Saudi Arabia, Hendrixson (2006) re-examined the specimens used by Levy and Amitai and found no significant differences from *A. crassicauda* except color variation. Hendrixson (2006) mentioned that *A. crassicauda* can be distinguished from *A. australis* by dark coloration and the granules on the ventrolateral carinae of metasomal segment V increase in size posteriorly. Al-Asmari et al. (2013: fig. 7) reported *A. australis* from Al-Medina Al-Munawwara region. The morphology and coloration are similar to *A. tihamicus* sp. nov., and this record probably was the new species. In molecular and morphometrical investigations, Alqahtani et al. (2022a, b, c) referred to the existence of three distinct clusters of *A. crassicauda* populations collected from different ecogeographical regions in Saudi Arabia, and the Arabian clade is placed as a basal clade to Turkey and Iran sequences. Ozkan et al. (2010) referred to two genetic groups of *A. crassicauda* found in Turkey, based on the 16S rRNA gene. Subsequently, Yağmur (2021) described a new species of *Androctonus* from the Şanlıurfa Province, Turkey, which had been previously identified as *A. crassicauda* (Fig. 16). Accordingly, seven species of the genus *Leiurus* Ehrenberg, 1828 in Arabia were described, transferred, or synonomized by Lowe et al. (2014) based on quantitative and qualitative morphological variations. Of these, *Leiurus brachycentrus* (Ehrenberg, 1829) occurs on the Tihamah Plain of western Yemen and southwestern Saudi Arabia. The observed diversity of scorpions is possibly a consequence of prominent geographical features, which contribute to an increased propensity for diversification in association with long-term processes such as geomorphological development and climatic cycles (Harzhauser et al. 2007; Lowe et al. 2014; Hou and Li 2018; Georgalis et al. 2020).

## Supplementary Material

XML Treatment for Androctonus tihamicus
